# Visualizing the Invisible—The Needs and Wishes of Childhood Cancer Survivors for Digitally Mediated Emotional Peer Support

**DOI:** 10.3390/curroncol29020108

**Published:** 2022-02-20

**Authors:** Stefan Nilsson, Ylva Hård af Segerstad, Maria Olsson

**Affiliations:** 1 Institute of Health and Care Sciences, Sahlgrenska Academy, University of Gothenburg, 405 30 Gothenburg, Sweden; 2 University of Gothenburg Centre for Person-Centred Care (GPCC), Sahlgrenska Academy, University of Gothenburg, 405 30 Gothenburg, Sweden; ylva.hard-af-segerstad@ait.gu.se (Y.H.a.S.); maria.olsson@gu.se (M.O.); 3 Department of Applied IT, University of Gothenburg, 417 56 Gothenburg, Sweden; 4 Institute of Clinical Sciences, Department of Oncology, University of Gothenburg, 405 30 Gothenburg, Sweden

**Keywords:** adolescents and young adults, childhood cancer long-term survivors, digital support, emotional support, qualitative research

## Abstract

This study aims to identify the needs and wishes of childhood cancer long-term survivors for digitally mediated emotional peer support. Survivors of childhood cancer (six men, seven women) aged 19–33, participated in semi-structured interviews (November–December 2020). Age of diagnosis ranged from 1 to 13 years. The interviews lasted between 45 and 85 min. A thematic analysis was used to identify three themes for needs: processing long-term complications of cancer treatment, processing psychosocial health and meeting others who share similar experiences; and another three themes reflecting wishes: digital tools for connecting with people who had had similar experiences, different modes of communication and a safe place with varying degrees of anonymity. The findings emphasized the needs and wishes of childhood cancer survivors to meet others who had had similar experiences using a digital tool that offered a secure place, with options for a variety of communication methods and levels of anonymity. Peer support can serve as an important complement to professional psychosocial support.

## 1. Introduction

Childhood cancer involves an intense treatment. In recent decades, improved treatment has contributed to increasing the survivor rate to over 80% in this population in the Nordic countries [1]. As a consequence, the number of individuals in society treated for childhood cancer has increased. However, intense cancer treatment causes several late complications, and about 60–80 percent of the individuals who have had childhood cancer experience them. Often, these complications do not appear until much later in life [2,3]. As an example, long-term survivors of childhood cancer can suffer from a number of mental health issues [4].

There is a need to understand how the risk of late complications and morbidity burden in this population can be decreased and how to optimize the well-being of childhood cancer survivors [3]. For example, previous research has found that adolescents and young adults (AYA) who survived their childhood cancer suffer from several physical symptoms, e.g., fatigue and pain, which might negatively affect their quality of life [5]. Fatigue has been shown to increase the risk of poor psychological functioning in survivors of childhood cancer [6]. In another study, AYA cancer survivors reported low satisfaction regarding their sexual function compared to controls [7]. In comparison to their peers, this population also focuses more on illness when they discuss their plans for the future [8]. Their position in society is negatively influenced, for example, with reduced socioeconomic attainment and fewer childhood cancer survivors graduating at college level and gaining full-time employment [9]. To follow up on these issues, routine psychological screening for all survivors of childhood cancer has been recommended, along with the establishment of follow-up clinics to meet these needs [10]. 

These results emphasize the importance of different types of support to optimize rehabilitation and well-being for AYA who have survived childhood cancer. A foundation of professional support is needed, with different forms of peer support as a valuable complement [11]. Peer support is likely to help childhood cancer survivors manage long-term complications in their daily life to a higher degree [12]. Peer support can be offered in a variety of forms, e.g., in patient organizations and meet-ups [13]. However, there is a lack of knowledge about how childhood cancer survivors’ needs and wishes are described when it comes to digitally mediated peer support. 

The results of previous research show that interventions based on face-to-face meetings have more positive effects compared to interventions conducted via the internet. Loneliness was identified as a possible explanation for this result, and personal contact could reduce the feeling of being alone [14]. In contrast, the results of another literature review showed no significant difference between personal contacts and internet-based interventions. The results could not show any benefits of one method over the other, and the interventions only gave moderate treatment effects. However, the authors still described these two types of interventions as being valuable for AYA long-term survivors of childhood cancer [15].

An alternative explanation for missing advantages of internet-based interventions might be the lack of studies about digital support. A systematic review of digital interventions for AYAs with cancer only found five studies that used mobile phone or tablet apps [16]. In developing digital tools for peer support, it is important to consider the goal of the intervention and the purpose of the technology. In addition, the suitability of the technology needs to be evaluated [17].

In Sweden, some digital media support exists for survivors of childhood cancer, for example, the app “War on cancer”. However, no studies have been found that describe users’ experience of these digital tools and whether or not they fulfill their needs and wishes.

This study aims to explore the needs and wishes of AYA childhood cancer long-term survivors for digitally mediated emotional peer support.

## 2. Materials and Methods

### 2.1. Design/Research Approach

This work was conducted as part of an innovation project (MyCode) developing interventions specific to the AYA cancer population. We used a qualitative study design to explore the needs and wishes of childhood cancer long-term survivors for digitally mediated emotional peer support. The consolidated criteria for reporting qualitative research (COREQ) 32-item checklist was used to report the research [18].

### 2.2. Participants

Survivors of childhood cancer in Sweden aged 16–35 were invited to participate in interviews inquiring into their wishes and needs for digitally mediated emotional peer support. Inclusion criteria for childhood cancer survivors were people who were diagnosed and had completed their treatment before the age of 18. Participants were recruited using purposive sampling through the follow-up clinics at two university hospitals and two national patient organizations during the summer and autumn of 2020. In order to include participants with a wide range of preferences, we invited both members of patient organizations and those without membership, as well as users and non-users of digital media. Invitations were distributed by postal letter and through the patient organizations’ webpages and digital newsletters. Exclusion criteria were inability to speak Swedish and severe cognitive disabilities, which make participating in dyadic interviews impossible. 

Thirteen survivors of childhood cancer (six men, seven women) aged 19–33 participated in the study. The participants had been diagnosed with a variety of cancer forms between the ages of 1–13: seven with leukemia, four with solid tumors and two with brain tumors. All the participants lived in Sweden, and our sample reflected the experience of AYAs in different geographical areas (Table 1).

### 2.3. Data Collection

We conducted semi-structured interviews between November and December 2020. All the participants received written information about the study and gave their oral and written consent for participation. Six interviews were dyadic [19], i.e., involving two participants. One interview was an individual interview. Additionally, each interview was led by a researcher (two females and a male, i.e., S.N., Y.H.a.S., M.O.) with a PhD in nursing, general linguistics or medicine. Two of the authors (S.N., M.O.) in the research group had extensive experience of pediatric oncology care. All interviews were observed by an AYA with cancer experience who was also trained in the research method. Patient involvement in the research process is necessary to interpret data successfully [20]. The interview guide contained some overarching categories., i.e., “access to social media”, “use of social media”, “topics covered in social media”, “desires for social media”. At the end of each interview, the observer summarized the interview, providing the interviewees with an opportunity to comment, clarify and correct, in what is known as a member check [21]. The interviews lasted between 45 and 85 min and were conducted online and recorded via the videoconferencing tool Zoom, which has been used extensively for research purposes [22]. The platform was chosen for being compliant with the Health Insurance Portability and Accountability Act (HIPPA) and for supporting video recording with local storage. The participants received a gift card as a token of thanks for their study participation.

### 2.4. Data Analysis

All the interviews were conducted in Swedish and were transcribed verbatim. The research group (i.e., the authors) read through the transcriptions separately several times. It was important that each researcher could independently interpret the data in the first step in order to fulfill credibility [21]. The data were coded with an increasing level of abstraction and interpretation to reach an understanding of the needs and wishes for digitally mediated peer support. Selective coding was performed. An inductive approach was adopted to develop the analysis collaboratively in order to identify patterns of themes in the data. In the final step, discussions about the themes were conducted until consensus was reached. The discussion focused on the purpose of the study and to strive for credibility in the analysis [21]. A thematic analysis was used to identify themes [23].

## 3. Results

All quotes were translated by a professional translator. The AYA childhood cancer survivors in our study identified a lack of existing resources for emotional support. For example, they did not have access to support that was appropriate for their individual needs. Long-term complications and psychosocial issues were frequently reported, along with the sense that few people in their existing social network fully understood their life situation. We identified three main themes in the interview data that expressed the AYA childhood cancer survivors’ needs and wishes for digitally mediated emotional support.

### 3.1. Needs

#### 3.1.1. Processing Long-Term Complications of Cancer Treatment

The participants expressed a need to process their thoughts on long-term complications. They described visible and invisible, as well as physical and psychological long-term complications and reported that they found these difficult to process with people who did not have similar experiences. Long-term complications were an urgent issue for the participants to discuss, but people around them did not always acknowledge and understand this need. There was sometimes a feeling that the AYAs who had survived childhood cancer should manage this themselves once they had finished treatment. In addition, our participants experienced that their long-term complications were not taken seriously and that others did not always recognize that a now healthy cancer survivor may still have problems post cancer. One participant summarized this experience by describing a reaction she had often met with, which had also been the experience of several other interviewees: “Well, you’re healthy now. Bye!” (Woman, 28 years).

A persistent narrative in our interview material was the need to emphasize long-term complications, not least because these complications may last a lifetime, even though they might not be visible to others. There was a need to reflect on this together with other cancer survivors who can support each other. The participants felt it could be difficult to talk with friends who had no cancer experience. “I’ve basically chosen not to tell too many of my friends that I’ve had it, for different reasons. For example, I’ve felt people have almost gotten a little scared [laughs] and just… and that’s when I’ve also felt I can’t talk about it because people find it incredibly hard.” (Woman, 22 years).

The participants expressed a need to exchange experiences with peers regarding late physical complications, so they could find strategies to handle their life situation. Long-term complications were sometimes found to contribute to difficulties in adapting to society. One dilemma that was mentioned as particularly challenging was the loss of fertility. “But then I have had side effects in terms of us not being able to have our own kids. I’ve had to go through that process at quite a late age.” (Man, 27 years).

#### 3.1.2. Processing Psychosocial Health

The participants emphasized psychosocial issues, such as anxiety and social phobia, as well as a lack of appropriate forums to address these issues. Social phobia was experienced as an obstacle, and some of the participants expressed a feeling of loneliness as a result. Social phobia in general was found to be very difficult to talk about, as it was experienced as a societal taboo. “It’s very hard to talk about social phobia in general and it’s very much a taboo. And particularly with people who don’t understand it, even though they have been on a similar journey, but it’s kind of not the main issue, so I’ve found it difficult to talk about.” (Woman, 28 years).

The participants described feeling particularly exposed to psychosocial problems during their teenage years. Nevertheless, even though they recognized this was a difficult time in life, the issues did not disappear with time, i.e., their needs remained. Some participants did not believe that professionals could help them, while others felt that professional support was appropriate. “I mean, like a psychologist—they are still trained to be able to care for people in a completely different way than I am. Say I might think... let’s say you get a negative comment, then I say: ‘Well, ignore them, they’re just idiots’, whereas a psychologist would have handled it in a completely different way, I think.” (Man, 28 years).

#### 3.1.3. Meeting Others Who Share Similar Experiences

The participants conveyed a need to meet others who shared similar experiences and who would be able to provide genuine understanding and support through their lived experiences. The participants described receiving lots of help from other cancer survivors who had gone through similar difficulties. Hearing how other cancer survivors had managed their situations inspired them to feel capable in relation to their own life situation. The participants said it was a relief to meet others with similar experiences and that it was sometimes easier to process issues together with them. “And most often you always find someone to... someone who has roughly the same diagnosis who you’ve been able to talk to and bond with and... I mean, it’s so important with this particular thing that... yes, having someone to talk to.” (Man, 25 years).

### 3.2. Wishes

The participants in the study expressed a wish for digitally mediated emotional support that would be available and accessible whenever they felt the need to talk about the effects of long-term complications or psychosocial issues. The digital format was seen as desirable, offering a variety of modes for communication to meet the needs of different individuals. One aspect that was highlighted as important was the need to feel secure in knowing that the communication platform could only be accessed by other cancer survivors.

#### 3.2.1. Digital Tools for Connecting with People Who Had Had Similar Experiences

The participants said it could be difficult to find others with similar experiences in their vicinity and that they often experienced loneliness after having cancer. Some mentioned that even their closest friends sometimes got tired of listening to all their stories, which was a reason for ceasing to talk to others about how they really felt. “My closest friends are a little bit tired of it but it’s kind of like you... it’s quite a big illness to just come out with, and if you come out with it to someone new, they often feel sorry for you and that’s not really what you want to get at.” (Man, 33 years).

A desire to find people with similar experiences was thus identified. However, our participants stated that it was not always easy to find similar others on the social media platforms that they were currently using. Some were not willing to expose themselves in the way required to find such contacts via social media, which is why they wanted a specific digital tool for cancer survivors that would facilitate finding individuals and groups that matched their needs in a secure environment. It was also suggested it would be valuable for the tool to be able to define age, diagnosis and interests in facilitating a search for similar others. “Yes, but like on Facebook it can be that you’re known by your name and surname, then you feel less secure [...] yes [it] can be linked to you in case you’re talking about sensitive things.” (Woman, 28 years) “So you also would have liked to be in touch with someone of the same age but there aren’t many teenagers affected. Most often they are younger.” (Woman, 28 years).

#### 3.2.2. Different Modes of Communication

Due to personal preferences or impairments, there was a wish for a variety of modes for communication, for example, communicating via text or video calls. Some participants preferred a computer screen rather than a mobile for video call due to visual impairment. People have different needs and preferences, and some find it easier to talk than to write, while for others it is the opposite. A common wish was to have a text-based, private chat function between two people. Another wish was the possibility to choose between one-to-one contacts or groups of different constellations. The topic of discussion steered the preferred mode of communication, i.e., it influenced whether the participants preferred to use text and chat with one person versus a group or use video. It was an individual choice whether to invite to a video chat or a text chat. “Yes, but you can have a few of you, but I imagine it’s one of those forums where you sit and talk on video like, then it can get a bit messy if there are too many of you. But a few are good.” (Woman, 19 years).

#### 3.2.3. A Safe Place with Varying Degrees of Anonymity

Feeling secure was identified as important for the participants. In developing a digitally mediated tool for emotional peer support, it is important to prevent random people from entering the forum. The participants wanted the forum to be only accessible to cancer survivors. They wanted some kind of secure login to access the platform and a moderator who could check the open forums and regulate things such as offensive language or notice if any of the participants seemed to need support. Being able to trust one another was expressed as a prerequisite for communication in a digital forum, including the possibility of sometimes remaining anonymous. Another option might be to start by using a nickname so that what you say is not directly identifiable to you as a person. This option was mentioned as something that would allow people to talk about sensitive things. However, others were hesitant about anonymity, as they thought it might decrease trust. “I think that to be able to build this platform, I think...because it’s quite sensitive and I know that if I joined it I might have wanted to be anonymous at first because even today I don’t talk openly about having had testicular cancer when lots of people are talking about it and asking.” (Man, 28 years) “When you’re anonymous the risk is, I think, it’s that maybe you...you don’t open up as much, you can’t give of yourself as much and so that might affect the rest of the conversation perhaps, that it can become more awkward.” (Man, 27 years).

## 4. Discussion

The main findings of this study stressed that long-term complications, both physical and psychological, influenced the AYA long-term survivors of childhood cancer. They felt a sense of loneliness in their immediate vicinity. Our findings are confirmed in another study showing that, although AYA survivors of childhood cancer were most focused on normality, they spent a lot of time dealing with their situation of being a survivor [24].

The participants expressed a need to get in touch with peers who share similar experiences of cancer effects. Contact with similar others can preferably take place on a digital platform that offers various modes of communication, such as text chat and video conferencing, and one-to-one or in groups. Our results contribute to the growing evidence that digital platforms play an important role in the support of mental and physical health outcomes among AYA long-term survivors of childhood cancer [25].

The participants in our study described physical long-term complications as important topics to discuss in peer support forums. For example, one relevant topic mentioned was fertility, and previous research has highlighted this as one of several important long-term physical complications. Other common long-term physical complications are endocrine dysfunction, neurological problems and heart failure [26,27]. 

Multiple physical long-term complications have contributed to presenteeism for AYA long-term survivors of childhood cancer. Their health problems mean they experience a deterioration in work performance and consequently spend more time working to compensate for this [28]. 

The participants in our study also identified long-term psychosocial complications as important, highlighting meeting others and sexuality as particular issues. In line with this, previous research has stressed sexuality as an urgent and necessary topic to discuss in this population [29,30,31]. AYA long-term survivors of childhood cancer are at risk of poor social outcomes and poor quality of life compared to healthy peers [32]. Consequently, there is a need for psychosocial screening in order to provide strategies for this group to cope with their burden [33,34,35]. The next step after screening could be to offer psychosocial support.

Psychosocial support is often divided into two different types: professional support and peer support. Our findings demonstrate that both types of support are important for AYA long-term survivors of childhood cancer. The participants emphasized a need for professional psychological support supplemented by contact with/support from other cancer survivors. Moreover, digitally mediated peer support was highlighted as a way to reach others. This is in line with previous research that supports the use of online groups, which can create a sense of community and belonging [36]. 

This study emphasized the need for both personal and online peer contact for AYA long-term survivors of childhood cancer. However, there is no consensus on whether personal contacts or internet-based interventions are the most beneficial solutions for offering psychosocial support [15]. There also seems to be a need for innovative solutions that facilitate different forms of peer support. Supportive interventions through peer support that involve peer relationships, self-esteem and cancer-related anxiety can probably promote positive psychosocial well-being [37].

In line with previous research [38], our findings emphasized that topics such as body image, sexuality and infertility are important to discuss in peer support forums. The participants in our study suggested that it would be valuable to be able to choose the level of anonymity and security when talking about sensitive topics, such as fertility. The digital format provides an opportunity to be anonymous in contrast to live physical contact, i.e., it provides a new and innovative way for AYA long-term survivors of childhood cancer to make contact with others. The importance of being able to vary the level of anonymity has also been highlighted by participants in a previous study [36]. In addition, previous research has emphasized the importance of being able to maintain a safe place in peer-to-peer web resources [39].

The participants were of the opinion that safety could be achieved by using a moderator of the peer support forum in order to control things such as abusive language or uncomfortable situations or to notice if any of the participants seem to be in need of support. The use of a moderator has been proved valuable in situations where participants need support and help [40].

Today, social media is probably the most common way for AYAs in general to communicate their feelings and well-being with peers and others [41]. In light of the current COVID-19 situation, socializing on the internet has increased, even among AYA long-term survivors of childhood cancer [32]. Most of these digital platforms are accessible and acceptable for most AYAs. However, more work is needed to evaluate what features of digitally mediated support are needed to be suitable in the context of peer support [17].

The present study has some limitations, such as the risk of selection bias. Our ambition was to recruit participants with a variety of experiences. Even so, we may have missed AYA long-term survivors of childhood cancer who do not like to be members of patient organizations. For the present study, we were also limited in only recruiting participants who were able to speak Swedish and thus missed the opportunity to get a multicultural view of this group’s needs and wishes for digitally mediated emotional support. The small sample size is also a limitation of this study, and it therefore needs to be repeated with a larger sample.

## 5. Conclusions

This study emphasized the needs and wishes of AYA long-term survivors of childhood cancer, and the participants emphasized the need to get in contact with others with similar experiences. Our findings further suggest that digital solutions should offer different forms of communication, such as text messaging, discussion forums and video conferencing. The digital format for peer support must also be perceived as safe for AYA long-term survivors of childhood cancer. A sense of security can be increased by using a login, different levels of anonymity and having a moderator to oversee/monitor the discussions. The next step will be to develop a tool for digitally mediated emotional peer support that fulfills the requirements of easy accessibility and high security.

## Figures and Tables

**Table 1:** Demographic data.

No.	Age	Gender	Work	Diagnosis	Years Since End of Treatment
1	19	Female	Student	Leukemia	17
2	22	Female	Student	Solid tumor	18
3	25	Male	Sick leave	Brain tumor	9
4	26	Male	Working	Brain tumor	8
5	24	Male	Working	Solid tumor	19
6	25	Female	Working	Leukemia	18
7	33	Male	Working	Leukemia	24
8	29	Female	Working	Leukemia	24
9	28	Female	Working	Leukemia	21
10	26	Female	Working	Leukemia	11
11	26	Female	Working	Solid tumor	11
12	28	Male	Working	Solid tumor	26
13	27	Male	Working	Leukemia	16

## Data Availability

The data used and/or analyzed during the current study are available from the corresponding author on reasonable request.
